# Triphenyl­phosphine oxide–2-(4-hydroxy­benzen­yl)-4,4,5,5-tetra­methyl­imidazolidine-1-oxyl 3-oxide (1/1)

**DOI:** 10.1107/S160053680903356X

**Published:** 2009-09-12

**Authors:** Lin-Lin Jing, Hai-Bo Wang, Xiao-Li Sun

**Affiliations:** a Department of Chemistry, School of Pharmacy, Fourth Military Medical University, Changle West Road 17, 710032 Xi-An, People’s Republic of China

## Abstract

The title compound, C_18_H_15_OP·C_13_H_17_N_2_O_3_, belongs to a series of mol­ecular systems based on triphenyl­phosphine oxide. The O atom of the oxide group acts as an acceptor for hydrogen bonds from –OH groups of the nitronyl nitroxide. The crystal structure is stabilized by O—H⋯O hydrogen bonds.

## Related literature

For related structures, see: Fuquen & Lechat (1992 ▶); Ng (2009 ▶). For hydrogen bonding, see: Etter (1990 ▶).
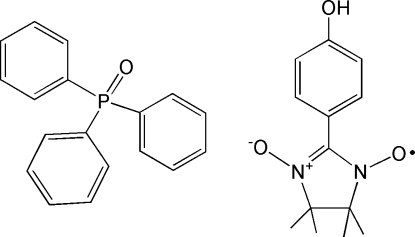


## Experimental

### 

#### Crystal data

C_18_H_15_OP·C_13_H_17_N_2_O_3_
*M* *_r_* = 527.56
Triclinic, 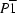

*a* = 8.8431 (11) Å
*b* = 12.0786 (15) Å
*c* = 13.9649 (16) Å
α = 86.386 (2)°
β = 82.724 (2)°
γ = 77.318 (2)°
*V* = 1442.6 (3) Å^3^
*Z* = 2
Mo *K*α radiation
μ = 0.13 mm^−1^
*T* = 296 K
0.39 × 0.28 × 0.16 mm

#### Data collection

Bruker APEXII CCD area-detector diffractometer
Absorption correction: multi-scan (*SADABS*; Bruker, 2000 ▶) *T*_min_ = 0.951, *T*_max_ = 0.979
7342 measured reflections
5071 independent reflections
2859 reflections with *I* > 2σ(*I*)
*R*_int_ = 0.024

#### Refinement

*R*[*F* ^2^ > 2σ(*F* ^2^)] = 0.045
*wR*(*F* ^2^) = 0.124
*S* = 1.08
5071 reflections
349 parameters
Hydrogen-atom parameters constrained
Δρ_max_ = 0.20 e Å^−3^
Δρ_min_ = −0.27 e Å^−3^

### 

Data collection: *APEX2* (Bruker, 2000 ▶); cell refinement: *SAINT* (Bruker, 2000 ▶); data reduction: *SAINT*; program(s) used to solve structure: *SHELXS97* (Sheldrick, 2008 ▶); program(s) used to refine structure: *SHELXL97* (Sheldrick, 2008 ▶); molecular graphics: *PLATON* (Spek, 2009 ▶); software used to prepare material for publication: *SHELXTL* (Sheldrick, 2008 ▶).

## Supplementary Material

Crystal structure: contains datablocks I, global. DOI: 10.1107/S160053680903356X/pb2003sup1.cif

Structure factors: contains datablocks I. DOI: 10.1107/S160053680903356X/pb2003Isup2.hkl

Additional supplementary materials:  crystallographic information; 3D view; checkCIF report

## Figures and Tables

**Table 1:** Hydrogen-bond geometry (Å, °)

*D*—H⋯*A*	*D*—H	H⋯*A*	*D*⋯*A*	*D*—H⋯*A*
O4—H4⋯O1^i^	0.82	1.82	2.633 (2)	171

Symmetry code: (i) 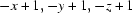.

**Fig. 1.**
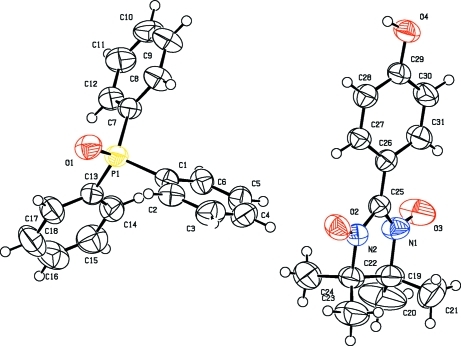
The molecular structure of the title compound (I), showing the atomic labelling scheme. The displacement ellipsoids are drawn at the 50% probability level.

**Fig. 2.**
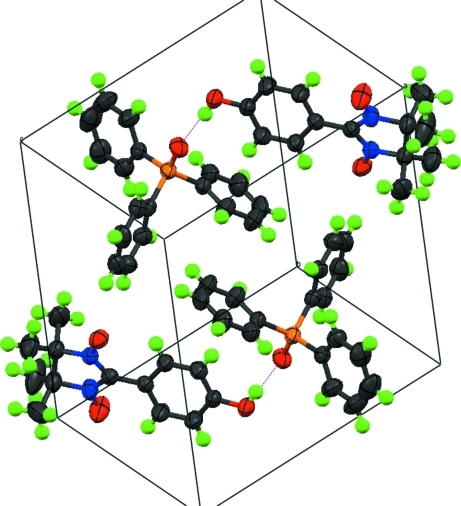
The packing of the title molecules (I), viewed down the a axis. Dotted lines indicate hydrogen bonds.

## supplementary crystallographic information

### Comment

In the title compound, the dihedral angle between the imidazole ring of the nitronyl nitroxide part and phenyl rings is 161.2°(2). The the nitronyl nitroxide and the triphenylphosphine oxide (TPPO) are linked by an O—H···O hydrogen bond. The O atom of the oxide group acts as an acceptor for hydrogen bonds from OH groups of the nitronyl nitroxide (Table1 and Fig.2).

### Experimental

Crystals of the title compound (I), were obtained by slow evaporation of equimolecular quantities of 4,4,5,5-tetramethyl-2-(4-hydroxybenzenyl)-imidazolidine-1-oxyl-3-oxide (2.49 g, 10.0 mmol) and triphenylphosphine oxide (2.78 g, 10.0 mmol) in 150 ml of dry acetonitrile. After three days, dark blue crystal of a good quality suitable for X-ray analysis were obtained.

### Refinement

In both structures all the H atoms were discernible in the difference Fourier maps. However, they were constrained by riding model approximation. *C*—*H*~methyl~=0.96 Å; *C*—Hãryl~=0.93 Å; *U*ĩso~*H*~methyl~ and *U*ĩso~Hãryl~ are 1.5 U ~eq~(C) and 1.2 U ~eq ~(C), respectively.

### Crystal data

**Table:**

C_18_H_15_OP·C_13_H_17_N_2_O_3_	*Z* = 2
*M**_r_* = 527.56	*F*(000) = 558
Triclinic, *P*1	*D*_x_ = 1.215 Mg m^−^^3^
Hall symbol: -P 1	Mo *K*α radiation, λ = 0.71073 Å
*a* = 8.8431 (11) Å	Cell parameters from 1409 reflections
*b* = 12.0786 (15) Å	θ = 2.4–20.6°
*c* = 13.9649 (16) Å	µ = 0.13 mm^−^^1^
α = 86.386 (2)°	*T* = 296 K
β = 82.724 (2)°	Block, blue
γ = 77.318 (2)°	0.39 × 0.28 × 0.16 mm
*V* = 1442.6 (3) Å^3^	

### Data collection

**Table:**

Bruker APEXII CCD area-detector diffractometer	5071 independent reflections
Radiation source: fine-focus sealed tube	2859 reflections with *I* > 2σ(*I*)
graphite	*R*_int_ = 0.024
φ and ω scans	θ_max_ = 25.1°, θ_min_ = 2.2°
Absorption correction: multi-scan (*SADABS*; Bruker, 2000)	*h* = −10→10
*T*_min_ = 0.951, *T*_max_ = 0.979	*k* = −9→14
7342 measured reflections	*l* = −16→16

### Refinement

**Table:**

Refinement on *F*^2^	Secondary atom site location: difference Fourier map
Least-squares matrix: full	Hydrogen site location: inferred from neighbouring sites
*R*[*F*^2^ > 2σ(*F*^2^)] = 0.045	H-atom parameters constrained
*wR*(*F*^2^) = 0.124	*w* = 1/[σ^2^(*F*_o_^2^) + (0.05*P*)^2^] where *P* = (*F*_o_^2^ + 2*F*_c_^2^)/3
*S* = 1.08	(Δ/σ)_max_ < 0.001
5071 reflections	Δρ_max_ = 0.20 e Å^−^^3^
349 parameters	Δρ_min_ = −0.27 e Å^−^^3^
0 restraints	Extinction correction: *SHELXL97* (Sheldrick, 2008), Fc^*^=kFc[1+0.001xFc^2^λ^3^/sin(2θ)]^-1/4^
Primary atom site location: structure-invariant direct methods	Extinction coefficient: 0.0101 (15)

### Special details

**Table:**

Geometry. All e.s.d.'s (except the e.s.d. in the dihedral angle between two l.s. planes) are estimated using the full covariance matrix. The cell e.s.d.'s are taken into account individually in the estimation of e.s.d.'s in distances, angles and torsion angles; correlations between e.s.d.'s in cell parameters are only used when they are defined by crystal symmetry. An approximate (isotropic) treatment of cell e.s.d.'s is used for estimating e.s.d.'s involving l.s. planes.
Refinement. Refinement of *F*^2^ against ALL reflections. The weighted *R*-factor *wR* and goodness of fit *S* are based on *F*^2^, conventional *R*-factors *R* are based on *F*, with *F* set to zero for negative *F*^2^. The threshold expression of *F*^2^ > σ(*F*^2^) is used only for calculating *R*-factors(gt) *etc*. and is not relevant to the choice of reflections for refinement. *R*-factors based on *F*^2^ are statistically about twice as large as those based on *F*, and *R*- factors based on ALL data will be even larger.

### Fractional atomic coordinates and isotropic or equivalent isotropic displacement parameters (Å^2^)

**Table:**

	*x*	*y*	*z*	*U*_iso_*/*U*_eq_	
P1	0.27687 (7)	0.23526 (6)	0.58436 (4)	0.0520 (2)	
N1	0.4223 (2)	0.70728 (19)	1.07295 (14)	0.0624 (6)	
N2	0.2585 (2)	0.69871 (16)	0.97237 (13)	0.0507 (5)	
O1	0.20487 (18)	0.22532 (16)	0.49552 (11)	0.0670 (5)	
O2	0.1903 (2)	0.70493 (15)	0.89585 (12)	0.0675 (5)	
O3	0.5469 (2)	0.7152 (2)	1.10822 (12)	0.0932 (7)	
O4	0.8088 (2)	0.85274 (18)	0.67430 (11)	0.0769 (6)	
H4	0.7939	0.8313	0.6224	0.115*	
C1	0.1934 (3)	0.3690 (2)	0.63862 (15)	0.0493 (6)	
C2	0.0567 (3)	0.4339 (2)	0.61134 (17)	0.0613 (7)	
H2	0.0079	0.4084	0.5645	0.074*	
C3	−0.0097 (3)	0.5378 (3)	0.6534 (2)	0.0743 (8)	
H3	−0.1017	0.5818	0.6342	0.089*	
C4	0.0613 (4)	0.5744 (3)	0.7230 (2)	0.0768 (9)	
H4A	0.0171	0.6435	0.7513	0.092*	
C5	0.1963 (4)	0.5105 (3)	0.75137 (19)	0.0719 (8)	
H5	0.2436	0.5360	0.7990	0.086*	
C6	0.2624 (3)	0.4091 (2)	0.70998 (17)	0.0599 (7)	
H6	0.3548	0.3661	0.7297	0.072*	
C7	0.4827 (3)	0.2292 (2)	0.56119 (16)	0.0536 (6)	
C8	0.5343 (3)	0.3206 (3)	0.51288 (19)	0.0714 (8)	
H8	0.4616	0.3849	0.4964	0.086*	
C9	0.6909 (4)	0.3179 (3)	0.4889 (2)	0.0862 (9)	
H9	0.7234	0.3799	0.4568	0.103*	
C10	0.7987 (3)	0.2232 (3)	0.5126 (2)	0.0855 (10)	
H10	0.9045	0.2211	0.4960	0.103*	
C11	0.7519 (3)	0.1319 (3)	0.5605 (2)	0.0829 (9)	
H11	0.8256	0.0683	0.5770	0.099*	
C12	0.5937 (3)	0.1347 (2)	0.58417 (19)	0.0670 (8)	
H12	0.5621	0.0721	0.6159	0.080*	
C13	0.2463 (3)	0.1266 (2)	0.67313 (18)	0.0531 (6)	
C14	0.2980 (3)	0.1184 (2)	0.76328 (19)	0.0669 (7)	
H14	0.3549	0.1695	0.7790	0.080*	
C15	0.2659 (4)	0.0350 (3)	0.8302 (2)	0.0864 (9)	
H15	0.3017	0.0296	0.8905	0.104*	
C16	0.1810 (4)	−0.0395 (3)	0.8072 (3)	0.0989 (11)	
H16	0.1578	−0.0947	0.8526	0.119*	
C17	0.1304 (4)	−0.0339 (3)	0.7190 (3)	0.0978 (11)	
H17	0.0738	−0.0855	0.7038	0.117*	
C18	0.1636 (3)	0.0491 (2)	0.6520 (2)	0.0737 (8)	
H18	0.1293	0.0526	0.5914	0.088*	
C19	0.2785 (3)	0.6952 (3)	1.13670 (18)	0.0708 (8)	
C20	0.3193 (4)	0.6162 (4)	1.2228 (2)	0.1369 (17)	
H20A	0.3829	0.5454	1.2006	0.205*	
H20B	0.2252	0.6028	1.2595	0.205*	
H20C	0.3757	0.6504	1.2629	0.205*	
C21	0.2011 (4)	0.8149 (3)	1.1698 (2)	0.1209 (14)	
H21A	0.2717	0.8439	1.2033	0.181*	
H21B	0.1077	0.8123	1.2123	0.181*	
H21C	0.1755	0.8636	1.1145	0.181*	
C22	0.1890 (3)	0.6513 (2)	1.06455 (17)	0.0610 (7)	
C23	0.0150 (3)	0.6928 (3)	1.0780 (2)	0.0964 (11)	
H23A	−0.0098	0.7744	1.0752	0.145*	
H23B	−0.0266	0.6657	1.1397	0.145*	
H23C	−0.0299	0.6650	1.0278	0.145*	
C24	0.2295 (4)	0.5222 (2)	1.0561 (2)	0.0959 (10)	
H24A	0.1819	0.5020	1.0035	0.144*	
H24B	0.1912	0.4871	1.1151	0.144*	
H24C	0.3407	0.4966	1.0443	0.144*	
C25	0.4003 (3)	0.7204 (2)	0.97831 (16)	0.0496 (6)	
C26	0.5070 (3)	0.7532 (2)	0.89941 (16)	0.0490 (6)	
C27	0.4924 (3)	0.7343 (2)	0.80365 (16)	0.0575 (7)	
H27	0.4142	0.6990	0.7906	0.069*	
C28	0.5908 (3)	0.7666 (2)	0.72880 (17)	0.0610 (7)	
H28	0.5783	0.7532	0.6657	0.073*	
C29	0.7083 (3)	0.8187 (2)	0.74579 (17)	0.0574 (7)	
C30	0.7242 (3)	0.8376 (3)	0.84031 (18)	0.0766 (9)	
H30	0.8022	0.8734	0.8530	0.092*	
C31	0.6268 (3)	0.8044 (2)	0.91523 (18)	0.0717 (8)	
H31	0.6413	0.8164	0.9783	0.086*	

### Atomic displacement parameters (Å^2^)

**Table:**

	*U*^11^	*U*^22^	*U*^33^	*U*^12^	*U*^13^	*U*^23^
P1	0.0468 (4)	0.0631 (5)	0.0482 (4)	−0.0173 (3)	−0.0024 (3)	−0.0048 (3)
N1	0.0557 (14)	0.0884 (18)	0.0443 (13)	−0.0201 (12)	−0.0038 (10)	−0.0002 (11)
N2	0.0525 (12)	0.0515 (13)	0.0485 (12)	−0.0131 (10)	−0.0037 (10)	−0.0021 (10)
O1	0.0613 (11)	0.0943 (14)	0.0500 (10)	−0.0218 (10)	−0.0099 (8)	−0.0124 (9)
O2	0.0683 (12)	0.0825 (14)	0.0614 (11)	−0.0342 (10)	−0.0140 (9)	0.0011 (10)
O3	0.0759 (13)	0.161 (2)	0.0569 (11)	−0.0530 (14)	−0.0201 (10)	0.0121 (12)
O4	0.0873 (13)	0.1037 (16)	0.0526 (10)	−0.0559 (12)	0.0090 (10)	−0.0096 (11)
C1	0.0469 (14)	0.0569 (16)	0.0454 (14)	−0.0163 (13)	−0.0044 (11)	0.0051 (12)
C2	0.0529 (16)	0.070 (2)	0.0592 (16)	−0.0133 (15)	−0.0035 (13)	0.0022 (15)
C3	0.0604 (18)	0.066 (2)	0.084 (2)	0.0040 (16)	0.0045 (16)	0.0036 (18)
C4	0.091 (2)	0.060 (2)	0.070 (2)	−0.0119 (19)	0.0190 (17)	−0.0071 (16)
C5	0.093 (2)	0.065 (2)	0.0616 (18)	−0.0270 (19)	−0.0041 (16)	−0.0083 (16)
C6	0.0645 (17)	0.0560 (18)	0.0608 (16)	−0.0154 (14)	−0.0100 (13)	−0.0002 (14)
C7	0.0500 (15)	0.0627 (18)	0.0499 (14)	−0.0185 (14)	0.0002 (11)	−0.0040 (13)
C8	0.0596 (18)	0.077 (2)	0.0799 (19)	−0.0239 (16)	−0.0042 (15)	0.0080 (16)
C9	0.070 (2)	0.097 (3)	0.097 (2)	−0.044 (2)	0.0083 (18)	0.004 (2)
C10	0.0465 (17)	0.112 (3)	0.095 (2)	−0.020 (2)	0.0190 (16)	−0.024 (2)
C11	0.0493 (17)	0.091 (2)	0.102 (2)	−0.0083 (17)	0.0054 (16)	−0.012 (2)
C12	0.0500 (16)	0.0664 (19)	0.0819 (19)	−0.0117 (15)	0.0070 (14)	−0.0144 (15)
C13	0.0438 (14)	0.0548 (17)	0.0609 (16)	−0.0131 (13)	0.0007 (12)	−0.0076 (13)
C14	0.0778 (19)	0.0577 (18)	0.0661 (18)	−0.0191 (15)	−0.0042 (15)	0.0006 (15)
C15	0.098 (2)	0.073 (2)	0.079 (2)	−0.009 (2)	−0.0025 (18)	0.0178 (18)
C16	0.085 (2)	0.060 (2)	0.140 (3)	−0.0107 (19)	0.010 (2)	0.028 (2)
C17	0.077 (2)	0.063 (2)	0.157 (3)	−0.0315 (19)	−0.003 (2)	0.003 (2)
C18	0.0634 (18)	0.0632 (19)	0.099 (2)	−0.0237 (16)	−0.0069 (16)	−0.0053 (18)
C19	0.0637 (18)	0.097 (2)	0.0549 (16)	−0.0298 (17)	0.0048 (14)	−0.0028 (17)
C20	0.113 (3)	0.248 (5)	0.064 (2)	−0.083 (3)	−0.0166 (19)	0.057 (3)
C21	0.111 (3)	0.144 (4)	0.114 (3)	−0.040 (3)	0.016 (2)	−0.070 (3)
C22	0.0602 (17)	0.070 (2)	0.0546 (15)	−0.0230 (15)	0.0022 (13)	0.0020 (14)
C23	0.066 (2)	0.136 (3)	0.091 (2)	−0.037 (2)	0.0027 (17)	−0.001 (2)
C24	0.124 (3)	0.062 (2)	0.097 (2)	−0.032 (2)	0.0199 (19)	0.0065 (18)
C25	0.0493 (15)	0.0511 (16)	0.0490 (15)	−0.0122 (12)	−0.0052 (12)	−0.0025 (12)
C26	0.0518 (15)	0.0522 (16)	0.0437 (14)	−0.0123 (12)	−0.0048 (11)	−0.0040 (12)
C27	0.0536 (15)	0.0720 (19)	0.0519 (15)	−0.0253 (14)	−0.0010 (12)	−0.0078 (14)
C28	0.0647 (17)	0.077 (2)	0.0461 (14)	−0.0242 (15)	−0.0061 (13)	−0.0073 (13)
C29	0.0635 (16)	0.0638 (18)	0.0488 (15)	−0.0272 (14)	0.0025 (13)	−0.0025 (13)
C30	0.083 (2)	0.106 (3)	0.0577 (17)	−0.0584 (19)	0.0008 (15)	−0.0151 (16)
C31	0.0812 (19)	0.101 (2)	0.0472 (15)	−0.0495 (18)	−0.0052 (14)	−0.0092 (15)

### Geometric parameters (Å, °)

**Table:**

P1—O1	1.4871 (15)	C14—H14	0.9300
P1—C13	1.789 (3)	C15—C16	1.368 (4)
P1—C7	1.794 (2)	C15—H15	0.9300
P1—C1	1.794 (2)	C16—C17	1.355 (4)
N1—O3	1.287 (2)	C16—H16	0.9300
N1—C25	1.355 (3)	C17—C18	1.382 (4)
N1—C19	1.486 (3)	C17—H17	0.9300
N2—O2	1.282 (2)	C18—H18	0.9300
N2—C25	1.349 (3)	C19—C20	1.517 (4)
N2—C22	1.491 (3)	C19—C21	1.530 (4)
O4—C29	1.356 (3)	C19—C22	1.542 (3)
O4—H4	0.8200	C20—H20A	0.9600
C1—C2	1.373 (3)	C20—H20B	0.9600
C1—C6	1.394 (3)	C20—H20C	0.9600
C2—C3	1.395 (3)	C21—H21A	0.9600
C2—H2	0.9300	C21—H21B	0.9600
C3—C4	1.366 (4)	C21—H21C	0.9600
C3—H3	0.9300	C22—C23	1.502 (3)
C4—C5	1.362 (4)	C22—C24	1.529 (4)
C4—H4A	0.9300	C23—H23A	0.9600
C5—C6	1.366 (3)	C23—H23B	0.9600
C5—H5	0.9300	C23—H23C	0.9600
C6—H6	0.9300	C24—H24A	0.9600
C7—C12	1.382 (3)	C24—H24B	0.9600
C7—C8	1.390 (4)	C24—H24C	0.9600
C8—C9	1.377 (4)	C25—C26	1.452 (3)
C8—H8	0.9300	C26—C31	1.384 (3)
C9—C10	1.370 (4)	C26—C27	1.397 (3)
C9—H9	0.9300	C27—C28	1.367 (3)
C10—C11	1.369 (4)	C27—H27	0.9300
C10—H10	0.9300	C28—C29	1.379 (3)
C11—C12	1.390 (3)	C28—H28	0.9300
C11—H11	0.9300	C29—C30	1.383 (3)
C12—H12	0.9300	C30—C31	1.367 (3)
C13—C18	1.374 (3)	C30—H30	0.9300
C13—C14	1.383 (3)	C31—H31	0.9300
C14—C15	1.381 (4)		
			
O1—P1—C13	111.66 (11)	C16—C17—H17	120.3
O1—P1—C7	112.90 (10)	C18—C17—H17	120.3
C13—P1—C7	108.14 (12)	C13—C18—C17	121.3 (3)
O1—P1—C1	110.81 (11)	C13—C18—H18	119.4
C13—P1—C1	107.11 (11)	C17—C18—H18	119.4
C7—P1—C1	105.88 (11)	N1—C19—C20	110.5 (2)
O3—N1—C25	125.7 (2)	N1—C19—C21	105.7 (2)
O3—N1—C19	121.27 (19)	C20—C19—C21	110.5 (3)
C25—N1—C19	112.7 (2)	N1—C19—C22	100.37 (19)
O2—N2—C25	126.60 (19)	C20—C19—C22	115.7 (3)
O2—N2—C22	119.92 (19)	C21—C19—C22	113.2 (2)
C25—N2—C22	113.11 (19)	C19—C20—H20A	109.5
C29—O4—H4	109.5	C19—C20—H20B	109.5
C2—C1—C6	118.3 (2)	H20A—C20—H20B	109.5
C2—C1—P1	120.01 (19)	C19—C20—H20C	109.5
C6—C1—P1	121.70 (19)	H20A—C20—H20C	109.5
C1—C2—C3	120.5 (2)	H20B—C20—H20C	109.5
C1—C2—H2	119.8	C19—C21—H21A	109.5
C3—C2—H2	119.8	C19—C21—H21B	109.5
C4—C3—C2	119.6 (3)	H21A—C21—H21B	109.5
C4—C3—H3	120.2	C19—C21—H21C	109.5
C2—C3—H3	120.2	H21A—C21—H21C	109.5
C5—C4—C3	120.5 (3)	H21B—C21—H21C	109.5
C5—C4—H4A	119.7	N2—C22—C23	110.8 (2)
C3—C4—H4A	119.7	N2—C22—C24	106.1 (2)
C4—C5—C6	120.1 (3)	C23—C22—C24	109.8 (3)
C4—C5—H5	119.9	N2—C22—C19	99.84 (19)
C6—C5—H5	119.9	C23—C22—C19	115.4 (2)
C5—C6—C1	120.9 (3)	C24—C22—C19	114.1 (2)
C5—C6—H6	119.5	C22—C23—H23A	109.5
C1—C6—H6	119.5	C22—C23—H23B	109.5
C12—C7—C8	117.9 (2)	H23A—C23—H23B	109.5
C12—C7—P1	123.0 (2)	C22—C23—H23C	109.5
C8—C7—P1	119.0 (2)	H23A—C23—H23C	109.5
C9—C8—C7	121.3 (3)	H23B—C23—H23C	109.5
C9—C8—H8	119.4	C22—C24—H24A	109.5
C7—C8—H8	119.4	C22—C24—H24B	109.5
C10—C9—C8	119.7 (3)	H24A—C24—H24B	109.5
C10—C9—H9	120.2	C22—C24—H24C	109.5
C8—C9—H9	120.2	H24A—C24—H24C	109.5
C11—C10—C9	120.5 (3)	H24B—C24—H24C	109.5
C11—C10—H10	119.7	N2—C25—N1	106.3 (2)
C9—C10—H10	119.7	N2—C25—C26	127.0 (2)
C10—C11—C12	119.7 (3)	N1—C25—C26	126.7 (2)
C10—C11—H11	120.2	C31—C26—C27	117.1 (2)
C12—C11—H11	120.2	C31—C26—C25	121.9 (2)
C7—C12—C11	120.9 (3)	C27—C26—C25	121.0 (2)
C7—C12—H12	119.5	C28—C27—C26	121.4 (2)
C11—C12—H12	119.5	C28—C27—H27	119.3
C18—C13—C14	118.2 (3)	C26—C27—H27	119.3
C18—C13—P1	118.5 (2)	C27—C28—C29	120.7 (2)
C14—C13—P1	123.3 (2)	C27—C28—H28	119.6
C15—C14—C13	120.7 (3)	C29—C28—H28	119.6
C15—C14—H14	119.7	O4—C29—C28	123.2 (2)
C13—C14—H14	119.7	O4—C29—C30	118.4 (2)
C16—C15—C14	119.5 (3)	C28—C29—C30	118.4 (2)
C16—C15—H15	120.2	C31—C30—C29	120.8 (2)
C14—C15—H15	120.2	C31—C30—H30	119.6
C17—C16—C15	120.9 (3)	C29—C30—H30	119.6
C17—C16—H16	119.5	C30—C31—C26	121.5 (2)
C15—C16—H16	119.5	C30—C31—H31	119.3
C16—C17—C18	119.4 (3)	C26—C31—H31	119.3
			
O1—P1—C1—C2	15.0 (2)	C25—N1—C19—C20	146.4 (3)
C13—P1—C1—C2	−107.0 (2)	O3—N1—C19—C21	79.6 (3)
C7—P1—C1—C2	137.75 (19)	C25—N1—C19—C21	−94.1 (3)
O1—P1—C1—C6	−166.18 (18)	O3—N1—C19—C22	−162.5 (2)
C13—P1—C1—C6	71.8 (2)	C25—N1—C19—C22	23.8 (3)
C7—P1—C1—C6	−43.4 (2)	O2—N2—C22—C23	−41.3 (3)
C6—C1—C2—C3	0.8 (4)	C25—N2—C22—C23	145.2 (2)
P1—C1—C2—C3	179.67 (19)	O2—N2—C22—C24	77.9 (3)
C1—C2—C3—C4	−0.7 (4)	C25—N2—C22—C24	−95.6 (2)
C2—C3—C4—C5	0.2 (4)	O2—N2—C22—C19	−163.3 (2)
C3—C4—C5—C6	0.3 (4)	C25—N2—C22—C19	23.1 (3)
C4—C5—C6—C1	−0.2 (4)	N1—C19—C22—N2	−25.6 (2)
C2—C1—C6—C5	−0.4 (4)	C20—C19—C22—N2	−144.4 (2)
P1—C1—C6—C5	−179.20 (19)	C21—C19—C22—N2	86.6 (3)
O1—P1—C7—C12	−105.0 (2)	N1—C19—C22—C23	−144.3 (2)
C13—P1—C7—C12	19.1 (2)	C20—C19—C22—C23	96.8 (3)
C1—P1—C7—C12	133.6 (2)	C21—C19—C22—C23	−32.1 (3)
O1—P1—C7—C8	71.1 (2)	N1—C19—C22—C24	87.1 (3)
C13—P1—C7—C8	−164.8 (2)	C20—C19—C22—C24	−31.7 (3)
C1—P1—C7—C8	−50.3 (2)	C21—C19—C22—C24	−160.7 (2)
C12—C7—C8—C9	−0.3 (4)	O2—N2—C25—N1	177.7 (2)
P1—C7—C8—C9	−176.6 (2)	C22—N2—C25—N1	−9.2 (3)
C7—C8—C9—C10	0.3 (5)	O2—N2—C25—C26	−1.2 (4)
C8—C9—C10—C11	−0.5 (5)	C22—N2—C25—C26	171.8 (2)
C9—C10—C11—C12	0.8 (5)	O3—N1—C25—N2	176.5 (2)
C8—C7—C12—C11	0.6 (4)	C19—N1—C25—N2	−10.1 (3)
P1—C7—C12—C11	176.8 (2)	O3—N1—C25—C26	−4.5 (4)
C10—C11—C12—C7	−0.9 (4)	C19—N1—C25—C26	168.8 (2)
O1—P1—C13—C18	−0.2 (2)	N2—C25—C26—C31	161.1 (2)
C7—P1—C13—C18	−125.0 (2)	N1—C25—C26—C31	−17.7 (4)
C1—P1—C13—C18	121.3 (2)	N2—C25—C26—C27	−18.9 (4)
O1—P1—C13—C14	−178.3 (2)	N1—C25—C26—C27	162.4 (2)
C7—P1—C13—C14	56.9 (2)	C31—C26—C27—C28	−1.0 (4)
C1—P1—C13—C14	−56.8 (2)	C25—C26—C27—C28	178.9 (2)
C18—C13—C14—C15	−0.5 (4)	C26—C27—C28—C29	0.2 (4)
P1—C13—C14—C15	177.6 (2)	C27—C28—C29—O4	−179.8 (2)
C13—C14—C15—C16	−0.5 (4)	C27—C28—C29—C30	0.0 (4)
C14—C15—C16—C17	1.2 (5)	O4—C29—C30—C31	−179.5 (3)
C15—C16—C17—C18	−0.7 (5)	C28—C29—C30—C31	0.6 (4)
C14—C13—C18—C17	1.0 (4)	C29—C30—C31—C26	−1.5 (5)
P1—C13—C18—C17	−177.2 (2)	C27—C26—C31—C30	1.6 (4)
C16—C17—C18—C13	−0.4 (5)	C25—C26—C31—C30	−178.3 (3)
O3—N1—C19—C20	−39.9 (4)		

### Hydrogen-bond geometry (Å, °)

**Table:**

*D*—H···*A*	*D*—H	H···*A*	*D*···*A*	*D*—H···*A*
O4—H4···O1^i^	0.82	1.82	2.633 (2)	171

Symmetry codes: (i) −*x*+1, −*y*+1, −*z*+1.
